# Diaqua­bis{5-carboxy-2-[(1*H*-1,2,4-triazol-1-yl)­meth­yl]-1*H*-imidazole-4-carboxyl­ato}­manganese(II)

**DOI:** 10.1107/S1600536810012626

**Published:** 2010-04-10

**Authors:** De-Gang Ding, Yan Tong

**Affiliations:** a Department of Quality Examination and Management, Zhengzhou College of Animal Husbandry Engineering, Zhengzhou, Henan 450011, People’s Republic of China

## Abstract

In the title compound, [Mn(C_8_H_6_N_5_O_4_)_2_(H_2_O)_2_], the Mn^II^ ion is situated on an inversion center and is six-coordinated by two N and two O atoms from two *L* ligands (H*L* = 2-[(1*H*-1,2,4-triazol-1-yl)meth­yl]-1*H*-imidazole-4,5-dicarboxylic acid) and two water mol­ecules in a distorted octa­hedral geometry. In ligand *L*, the imidazole and triazole rings form a dihedral angle of 74.25 (8)°. Mol­ecules are assembled into a three-dimensional structure *via* inter­molecular O—H⋯O, O—H⋯N and N—H⋯N hydrogen-bonds, and π–π inter­actions with a short distance of 3.665 (2) Å between the centroids of the imidazole and triazole rings of neighbouring mol­ecules.

## Related literature

For related structures, see: Lee *et al.* (2005 ▶); Ouellette *et al.* (2007 ▶); Won *et al.* (2007 ▶).
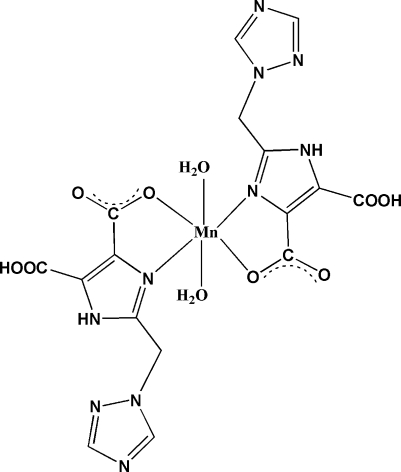


## Experimental

### 

#### Crystal data

[Mn(C_8_H_6_N_5_O_4_)_2_(H_2_O)_2_]
*M* *_r_* = 563.33
Monoclinic, 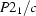

*a* = 7.730 (2) Å
*b* = 14.498 (3) Å
*c* = 11.588 (4) Å
β = 125.70 (2)°
*V* = 1054.6 (5) Å^3^
*Z* = 2
Mo *K*α radiation
μ = 0.71 mm^−1^
*T* = 293 K
0.20 × 0.15 × 0.10 mm

#### Data collection

Rigaku Mercury CCD area-detector diffractometer
Absorption correction: multi-scan (*CrystalClear*; Rigaku, 2000 ▶) *T*_min_ = 0.871, *T*_max_ = 0.933
11281 measured reflections
2074 independent reflections
1974 reflections with *I* > 2σ(*I*)
*R*_int_ = 0.033

#### Refinement

*R*[*F* ^2^ > 2σ(*F* ^2^)] = 0.038
*wR*(*F* ^2^) = 0.077
*S* = 1.05
2074 reflections
169 parameters
H-atom parameters constrained
Δρ_max_ = 0.25 e Å^−3^
Δρ_min_ = −0.25 e Å^−3^

### 

Data collection: *CrystalClear* (Rigaku, 2000 ▶); cell refinement: *CrystalClear*; data reduction: *CrystalClear*; program(s) used to solve structure: *SHELXS97* (Sheldrick, 2008 ▶); program(s) used to refine structure: *SHELXL97* (Sheldrick, 2008 ▶); molecular graphics: *SHELXTL* (Sheldrick, 2008 ▶); software used to prepare material for publication: *SHELXTL*.

## Supplementary Material

Crystal structure: contains datablocks global, I. DOI: 10.1107/S1600536810012626/cv2709sup1.cif

Structure factors: contains datablocks I. DOI: 10.1107/S1600536810012626/cv2709Isup2.hkl

Additional supplementary materials:  crystallographic information; 3D view; checkCIF report

## Figures and Tables

**Table 1:** Hydrogen-bond geometry (Å, °)

*D*—H⋯*A*	*D*—H	H⋯*A*	*D*⋯*A*	*D*—H⋯*A*
O3—H3*C*⋯O2	0.98	1.50	2.483 (2)	178
O5—H5*B*⋯N2^i^	0.78	2.18	2.878 (2)	149
O5—H5*C*⋯O4^ii^	0.80	1.98	2.755 (2)	162
N5—H5*A*⋯N3^iii^	0.86	1.96	2.811 (2)	169

Symmetry codes: (i) 
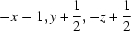
; (ii) 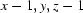; (iii) 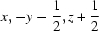.

**Fig. 1.**
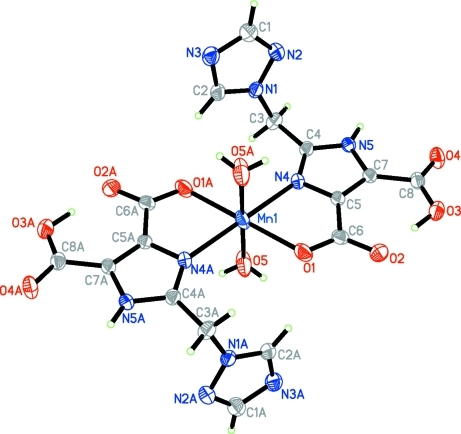
The molecular structure of (I) showing 50% probability displacement ellipsoids and the atom-numbering [symmetry code: (A) - x - 1, - y, - z].

## supplementary crystallographic information

### Comment

Multidentate ligands containing rich coordination sites (N and/or O donors) are often employed to produce polymeric networks with structural diversity owing to their various coordination modes (Lee *et al.*, 2005; Ouellette *et al.*, 2007; Won *et al.*, 2007). As ligands with multiple coordination sites, 1,2,4-triazole and its derivatives have been shown to be good organic linkers in generation of structurally versatile metal-organic frameworks since it can bridge different metal centers to afford coordination polymers that exhibit extraordinary structural diversity and facile accessibility of functionalized materials. We selected a ligand containing 1,2,4-triazole, imidazole, and carboxylate groups, 2-[(1*H*-1,2,4-triazol-1-yl)methyl]-1*H*-imidazole-4,5-dicarboxylic acid, to study its coordination chemistry. As a result, we report herein the crystal structure of the title compound (I).

In (I), Mn^II^ ion located on an inversion center is six–coordinated by two imidazole nitrogen atoms (N4), two carboxylate group oxygen atoms (O1) from two ligands, and two water oxygen atoms (Fig. 1). The coordination bond lengths Mn—N and Mn—O are 2.248 (1), 2.186 (1) Å and 2.188 (2) Å, respectively. The coordination geometry around Mn^II^ is a distorted octahedron - the Mn^II^ coordination angles are in the range from 75.75 (6)° to 180.00 (1)°. Each *L* acts as a bidentate ligand.

In the crystal structure, the intra- and intermolecular hydrogen bonds (Table 1) and π–π interactions with short distance of 3.665 (2) Å between the centroids of imidazole and triazole rings from the neighbouring molecules consolidate the crystal packing.

### Experimental

All solvents and chemicals were of analytical grade and were used without further purification. The compound [Mn*L*_2_(H_2_O)_2_] was synthesized as follows: 2-[(1*H*-1,2,4-triazol-1-yl)methyl]-1*H*-imidazole-4,5-dicarboxylic acid (1.0 mmol) was added to 5 cm^3^ water and the resulting solution was adjusted pH to 7.0 by NaOH aqueous. Then MnCl_2_(0.5 mmol) was added to the above solution, and the mixture was stirred for 30 min and filtered. After one days, pink single crystals suitable for X-ray analysis were obtained. Analysis calculated (%) for C_16_H_16_MnN_10_O_10_: C 34.12, H 2.86, N 24.87; found (%): C 34.23, H 2.65, N 24.75.

### Refinement

The H atoms were included in calculated positions and treated as riding atoms: C—H = 0.93 Å for the triazole, 0.97 Å for the methylene H atoms, O—H = 0.79 Å for water molecule, 0.98 Å for carboxylic acid, and N—H = 0.86 Å for the imidazole, with U_iso_(H) = 1.5U_eq_(parent O-atom) and 1.2U_eq_(parent N-atom and C-atom).

### Crystal data

**Table:**

[Mn(C_8_H_6_N_5_O_4_)_2_(H_2_O)_2_]	*F*(000) = 574
*M**_r_* = 563.33	*D*_x_ = 1.774 Mg m^−^^3^
Monoclinic, *P*2_1_/*c*	Mo *K*α radiation, λ = 0.71073 Å
Hall symbol: -P 2ybc	Cell parameters from 3276 reflections
*a* = 7.730 (2) Å	θ = 2.6–30.8°
*b* = 14.498 (3) Å	µ = 0.71 mm^−^^1^
*c* = 11.588 (4) Å	*T* = 293 K
β = 125.70 (2)°	Prism, pink
*V* = 1054.6 (5) Å^3^	0.20 × 0.15 × 0.10 mm
*Z* = 2	

### Data collection

**Table:**

Rigaku Mercury CCD area-detector diffractometer	2074 independent reflections
Radiation source: fine-focus sealed tube	1974 reflections with *I* > 2σ(*I*)
graphite	*R*_int_ = 0.033
ω scans	θ_max_ = 26.0°, θ_min_ = 2.6°
Absorption correction: multi-scan (*CrystalClear*; Rigaku, 2000)	*h* = −9→9
*T*_min_ = 0.871, *T*_max_ = 0.933	*k* = −17→17
11281 measured reflections	*l* = −13→14

### Refinement

**Table:**

Refinement on *F*^2^	Primary atom site location: structure-invariant direct methods
Least-squares matrix: full	Secondary atom site location: difference Fourier map
*R*[*F*^2^ > 2σ(*F*^2^)] = 0.038	Hydrogen site location: inferred from neighbouring sites
*wR*(*F*^2^) = 0.077	H-atom parameters constrained
*S* = 1.05	*w* = 1/[σ^2^(*F*_o_^2^) + (0.0245*P*)^2^ + 0.947*P*] where *P* = (*F*_o_^2^ + 2*F*_c_^2^)/3
2074 reflections	(Δ/σ)_max_ < 0.001
169 parameters	Δρ_max_ = 0.25 e Å^−^^3^
0 restraints	Δρ_min_ = −0.25 e Å^−^^3^

### Special details

**Table:**

Geometry. Bond distances, angles etc. have been calculated using the rounded fractional coordinates. All su's are estimated from the variances of the (full) variance-covariance matrix. The cell esds are taken into account in the estimation of distances, angles and torsion angles.
Refinement. Refinement of *F*^2^ against ALL reflections. The weighted *R*-factor *wR* and goodness of fit *S* are based on *F*^2^, conventional *R*-factors *R* are based on *F*, with *F* set to zero for negative *F*^2^. The threshold expression of *F*^2^ > σ(*F*^2^) is used only for calculating *R*-factors(gt) *etc*. and is not relevant to the choice of reflections for refinement. *R*-factors based on *F*^2^ are statistically about twice as large as those based on *F*, and *R*- factors based on ALL data will be even larger.

### Fractional atomic coordinates and isotropic or equivalent isotropic displacement parameters (Å^2^)

**Table:**

	*x*	*y*	*z*	*U*_iso_*/*U*_eq_	
Mn1	−0.5000	0.0000	0.0000	0.02963 (14)	
O1	−0.2806 (2)	0.11091 (10)	0.13249 (15)	0.0333 (4)	
O2	−0.0902 (2)	0.17418 (10)	0.34848 (15)	0.0319 (3)	
O3	−0.0253 (2)	0.14770 (10)	0.58250 (15)	0.0328 (4)	
H3C	−0.0491	0.1596	0.4908	0.049*	
O4	−0.1284 (2)	0.04886 (11)	0.67687 (15)	0.0353 (4)	
O5	−0.7693 (3)	0.08739 (13)	−0.05545 (17)	0.0516 (5)	
H5B	−0.7549	0.1335	−0.0155	0.077*	
H5C	−0.8757	0.0886	−0.1346	0.077*	
N1	−0.4778 (3)	−0.24271 (11)	0.21460 (17)	0.0247 (4)	
N2	−0.3256 (3)	−0.29594 (12)	0.32424 (18)	0.0316 (4)	
N3	−0.3707 (3)	−0.33526 (13)	0.12013 (18)	0.0318 (4)	
N4	−0.4235 (3)	−0.02708 (11)	0.21633 (16)	0.0229 (4)	
N5	−0.3572 (3)	−0.05521 (11)	0.42541 (16)	0.0221 (4)	
H5A	−0.3594	−0.0820	0.4907	0.026*	
C1	−0.2671 (4)	−0.34974 (16)	0.2612 (2)	0.0337 (5)	
H1A	−0.1620	−0.3945	0.3101	0.040*	
C2	−0.5007 (4)	−0.26688 (14)	0.0956 (2)	0.0290 (5)	
H2A	−0.5952	−0.2394	0.0074	0.035*	
C3	−0.5880 (3)	−0.16935 (14)	0.2336 (2)	0.0283 (5)	
H3A	−0.6208	−0.1902	0.2984	0.034*	
H3B	−0.7215	−0.1559	0.1430	0.034*	
C4	−0.4575 (3)	−0.08370 (13)	0.2908 (2)	0.0219 (4)	
C5	−0.2913 (3)	0.04042 (13)	0.30983 (19)	0.0208 (4)	
C6	−0.2149 (3)	0.11379 (14)	0.2602 (2)	0.0249 (4)	
C7	−0.2507 (3)	0.02413 (13)	0.44029 (19)	0.0212 (4)	
C8	−0.1284 (3)	0.07527 (14)	0.5765 (2)	0.0252 (4)	

### Atomic displacement parameters (Å^2^)

**Table:**

	*U*^11^	*U*^22^	*U*^33^	*U*^12^	*U*^13^	*U*^23^
Mn1	0.0324 (3)	0.0372 (3)	0.0167 (2)	−0.0015 (2)	0.0129 (2)	0.00040 (18)
O1	0.0379 (9)	0.0365 (9)	0.0233 (8)	−0.0041 (7)	0.0166 (7)	0.0066 (6)
O2	0.0335 (8)	0.0273 (8)	0.0316 (8)	−0.0063 (6)	0.0172 (7)	−0.0006 (6)
O3	0.0347 (8)	0.0307 (8)	0.0279 (8)	−0.0076 (7)	0.0153 (7)	−0.0074 (6)
O4	0.0384 (9)	0.0452 (10)	0.0207 (8)	−0.0050 (7)	0.0162 (7)	−0.0059 (7)
O5	0.0416 (10)	0.0684 (13)	0.0262 (9)	0.0132 (9)	0.0093 (8)	−0.0137 (8)
N1	0.0317 (9)	0.0216 (9)	0.0235 (9)	−0.0040 (7)	0.0176 (8)	−0.0033 (7)
N2	0.0376 (10)	0.0316 (10)	0.0224 (9)	−0.0003 (8)	0.0156 (8)	0.0009 (7)
N3	0.0410 (11)	0.0312 (10)	0.0293 (10)	−0.0014 (8)	0.0240 (9)	−0.0032 (8)
N4	0.0250 (8)	0.0240 (9)	0.0175 (8)	−0.0010 (7)	0.0111 (7)	−0.0016 (6)
N5	0.0268 (9)	0.0229 (9)	0.0185 (8)	0.0000 (7)	0.0144 (7)	0.0004 (6)
C1	0.0351 (12)	0.0332 (12)	0.0300 (12)	0.0032 (10)	0.0174 (10)	0.0006 (9)
C2	0.0410 (12)	0.0249 (11)	0.0227 (10)	−0.0040 (9)	0.0196 (10)	−0.0022 (8)
C3	0.0330 (11)	0.0243 (11)	0.0332 (11)	−0.0039 (9)	0.0225 (10)	−0.0057 (9)
C4	0.0230 (10)	0.0213 (10)	0.0217 (10)	0.0002 (8)	0.0133 (8)	−0.0016 (8)
C5	0.0210 (9)	0.0201 (9)	0.0189 (9)	0.0021 (8)	0.0102 (8)	0.0004 (8)
C6	0.0234 (10)	0.0238 (10)	0.0240 (10)	0.0033 (8)	0.0119 (9)	0.0046 (8)
C7	0.0207 (9)	0.0214 (10)	0.0188 (9)	0.0019 (8)	0.0100 (8)	0.0004 (7)
C8	0.0227 (10)	0.0274 (11)	0.0211 (10)	0.0034 (8)	0.0102 (8)	−0.0025 (8)

### Geometric parameters (Å, °)

**Table:**

Mn1—O5^i^	2.1886 (17)	N2—C1	1.316 (3)
Mn1—O5	2.1886 (17)	N3—C2	1.319 (3)
Mn1—O1^i^	2.1862 (16)	N3—C1	1.353 (3)
Mn1—O1	2.1862 (16)	N4—C4	1.324 (2)
Mn1—N4	2.2489 (18)	N4—C5	1.373 (2)
Mn1—N4^i^	2.2489 (18)	N5—C4	1.339 (2)
O1—C6	1.254 (2)	N5—C7	1.366 (2)
O2—C6	1.262 (2)	N5—H5A	0.8600
O3—C8	1.296 (3)	C1—H1A	0.9300
O3—H3C	0.9817	C2—H2A	0.9300
O4—C8	1.224 (2)	C3—C4	1.489 (3)
O5—H5B	0.7826	C3—H3A	0.9700
O5—H5C	0.7987	C3—H3B	0.9700
N1—C2	1.331 (3)	C5—C7	1.373 (3)
N1—N2	1.360 (2)	C5—C6	1.486 (3)
N1—C3	1.458 (3)	C7—C8	1.481 (3)
			
O5^i^—Mn1—O5	180.00 (12)	C4—N5—H5A	126.2
O5^i^—Mn1—O1^i^	89.74 (7)	C7—N5—H5A	126.2
O5—Mn1—O1^i^	90.26 (7)	N2—C1—N3	115.2 (2)
O5^i^—Mn1—O1	90.26 (7)	N2—C1—H1A	122.4
O5—Mn1—O1	89.74 (7)	N3—C1—H1A	122.4
O1^i^—Mn1—O1	180.00 (10)	N3—C2—N1	110.49 (19)
O5^i^—Mn1—N4	89.15 (6)	N3—C2—H2A	124.8
O5—Mn1—N4	90.85 (6)	N1—C2—H2A	124.8
O1^i^—Mn1—N4	104.25 (6)	N1—C3—C4	111.81 (16)
O1—Mn1—N4	75.75 (6)	N1—C3—H3A	109.3
O5^i^—Mn1—N4^i^	90.85 (6)	C4—C3—H3A	109.3
O5—Mn1—N4^i^	89.15 (6)	N1—C3—H3B	109.3
O1^i^—Mn1—N4^i^	75.75 (6)	C4—C3—H3B	109.3
O1—Mn1—N4^i^	104.25 (6)	H3A—C3—H3B	107.9
N4—Mn1—N4^i^	180.00 (11)	N4—C4—N5	111.56 (17)
C6—O1—Mn1	117.87 (13)	N4—C4—C3	124.59 (17)
C8—O3—H3C	111.3	N5—C4—C3	123.85 (18)
Mn1—O5—H5B	122.5	N4—C5—C7	109.45 (17)
Mn1—O5—H5C	121.0	N4—C5—C6	119.06 (16)
H5B—O5—H5C	110.9	C7—C5—C6	131.47 (18)
C2—N1—N2	109.77 (17)	O1—C6—O2	124.89 (19)
C2—N1—C3	127.77 (18)	O1—C6—C5	117.01 (18)
N2—N1—C3	122.43 (16)	O2—C6—C5	118.10 (17)
C1—N2—N1	102.01 (17)	N5—C7—C5	105.79 (16)
C2—N3—C1	102.53 (18)	N5—C7—C8	121.25 (17)
C4—N4—C5	105.50 (15)	C5—C7—C8	132.93 (18)
C4—N4—Mn1	144.26 (13)	O4—C8—O3	122.92 (18)
C5—N4—Mn1	110.24 (12)	O4—C8—C7	120.16 (19)
C4—N5—C7	107.68 (16)	O3—C8—C7	116.91 (18)
			
O5^i^—Mn1—O1—C6	−90.63 (15)	C5—N4—C4—C3	−178.15 (18)
O5—Mn1—O1—C6	89.37 (15)	Mn1—N4—C4—C3	2.4 (4)
O1^i^—Mn1—O1—C6	178 (100)	C7—N5—C4—N4	−0.3 (2)
N4—Mn1—O1—C6	−1.57 (14)	C7—N5—C4—C3	178.78 (18)
N4^i^—Mn1—O1—C6	178.43 (14)	N1—C3—C4—N4	73.8 (2)
C2—N1—N2—C1	−0.1 (2)	N1—C3—C4—N5	−105.2 (2)
C3—N1—N2—C1	−178.39 (18)	C4—N4—C5—C7	−1.2 (2)
O5^i^—Mn1—N4—C4	−87.9 (2)	Mn1—N4—C5—C7	178.46 (12)
O5—Mn1—N4—C4	92.1 (2)	C4—N4—C5—C6	177.62 (17)
O1^i^—Mn1—N4—C4	1.6 (2)	Mn1—N4—C5—C6	−2.7 (2)
O1—Mn1—N4—C4	−178.4 (2)	Mn1—O1—C6—O2	−179.15 (15)
N4^i^—Mn1—N4—C4	171 (100)	Mn1—O1—C6—C5	0.6 (2)
O5^i^—Mn1—N4—C5	92.68 (13)	N4—C5—C6—O1	1.6 (3)
O5—Mn1—N4—C5	−87.32 (13)	C7—C5—C6—O1	−179.9 (2)
O1^i^—Mn1—N4—C5	−177.81 (12)	N4—C5—C6—O2	−178.64 (17)
O1—Mn1—N4—C5	2.19 (12)	C7—C5—C6—O2	−0.1 (3)
N4^i^—Mn1—N4—C5	−9(100)	C4—N5—C7—C5	−0.4 (2)
N1—N2—C1—N3	−0.3 (2)	C4—N5—C7—C8	177.95 (17)
C2—N3—C1—N2	0.6 (3)	N4—C5—C7—N5	1.0 (2)
C1—N3—C2—N1	−0.7 (2)	C6—C5—C7—N5	−177.59 (19)
N2—N1—C2—N3	0.6 (2)	N4—C5—C7—C8	−177.1 (2)
C3—N1—C2—N3	178.69 (18)	C6—C5—C7—C8	4.3 (4)
C2—N1—C3—C4	−97.8 (2)	N5—C7—C8—O4	−3.0 (3)
N2—N1—C3—C4	80.2 (2)	C5—C7—C8—O4	174.9 (2)
C5—N4—C4—N5	0.9 (2)	N5—C7—C8—O3	177.23 (17)
Mn1—N4—C4—N5	−178.52 (16)	C5—C7—C8—O3	−4.9 (3)

Symmetry codes: (i) −*x*−1, −*y*, −*z*.

### Hydrogen-bond geometry (Å, °)

**Table:**

*D*—H···*A*	*D*—H	H···*A*	*D*···*A*	*D*—H···*A*
O3—H3C···O2	0.98	1.50	2.483 (2)	178
O5—H5B···N2^ii^	0.78	2.18	2.878 (2)	149
O5—H5C···O4^iii^	0.80	1.98	2.755 (2)	162
N5—H5A···N3^iv^	0.86	1.96	2.811 (2)	169

Symmetry codes: (ii) −*x*−1, *y*+1/2, −*z*+1/2; (iii) *x*−1, *y*, *z*−1; (iv) *x*, −*y*−1/2, *z*+1/2.
